# Biochemical and Electrophysiological Characterization of Two Sea Anemone Type 1 Potassium Toxins from a Geographically Distant Population of *Bunodosoma caissarum*

**DOI:** 10.3390/md11030655

**Published:** 2013-03-06

**Authors:** Diego J. B. Orts, Steve Peigneur, Bruno Madio, Juliana S. Cassoli, Gabriela G. Montandon, Adriano M. C. Pimenta, José E. P. W. Bicudo, José C. Freitas, André J. Zaharenko, Jan Tytgat

**Affiliations:** 1 Department of Physiology, Institute of Biosciences, University of São Paulo, São Paulo, SP, 05508-090, Brazil; E-Mails: diego.orts@usp.br (D.J.B.O.); brunomadio@usp.br (B.M.); jebicudo@usp.br (J.E.P.W.B.); freitas_jose@rocketmail.com (J.C.F.); 2 Center of Marine Biology, University of São Paulo, São Sebastião, SP, 11600-000, Brazil; 3 Laboratory of Toxicology, University of Leuven (K.U. Leuven), Campus Gasthuisberg O&N2, Herestraat 49, P.O. Box 922, 3000 Leuven, Belgium; E-Mail: steve.peigneur@pharm.kuleuven.be; 4 Laboratory of Venoms and Animals Toxins, Institute of Biological Sciences, Federal University of Minas Gerais, Belo Horizonte, MG, 31270-901, Brazil; E-Mails: jscassoli@ufmg.br (J.S.C.); ggmufmg@gmail.com (G.G.M.); apimenta@icb.ufmg.br (A.M.C.P.); 5 Laboratorio de Genetica, Instituto Butantan, São Paulo, SP, 05503-900, Brazil

**Keywords:** sea anemone, *Bunodosoma caissarum*, neurotoxins, voltage-gated potassium channels, two-electrode voltage-clamp, *Xenopus laevis*, intraspecific venom variation, Saint Peter and Saint Paul Archipelago

## Abstract

Sea anemone (Cnidaria, Anthozoa) venom is an important source of bioactive compounds used as tools to study the pharmacology and structure-function of voltage-gated K^+^ channels (K_V_). These neurotoxins can be divided into four different types, according to their structure and mode of action. In this work, for the first time, two toxins were purified from the venom of *Bunodosoma caissarum* population from Saint Peter and Saint Paul Archipelago, Brazil. Sequence alignment and phylogenetic analysis reveals that BcsTx1 and BcsTx2 are the newest members of the sea anemone type 1 potassium channel toxins. Their functional characterization was performed by means of a wide electrophysiological screening on 12 different subtypes of K_V_ channels (K_V_1.1–K_V_1.6; K_V_2.1; K_V_3.1; K_V_4.2; K_V_4.3; *h*ERG and *Shaker* IR). BcsTx1 shows a high affinity for rKv1.2 over rKv1.6, hKv1.3, *Shaker* IR and rKv1.1, while Bcstx2 potently blocked rKv1.6 over hKv1.3, rKv1.1, *Shaker* IR and rKv1.2. Furthermore, we also report for the first time a venom composition and biological activity comparison between two geographically distant populations of sea anemones.

## 1. Introduction

As the most ancient venomous animals on Earth, cnidarians (classes Anthozoa, Scyphozoa, Cubozoa and Hydrozoa) have evolved a large amount of pore-forming toxins, phospholipases A_2_, protease inhibitors, neurotoxins and toxic secondary metabolites [1,2,3,4,5,6]. The biological and ecological roles of these toxins present in cnidarians venom are (1) immobilization and death of the prey, (2) defense against predators and (3) intra- and inter-specific competition [7,8,9,10,11]. Sea anemones (Anthozoa, Actiniaria) are a well-known pharmacological source of a large number of neurotoxins acting upon a diverse panel of ion channels, such as voltage-gated sodium and potassium channels. Toxins that target sodium channels are the best-studied group, with more than 100 described toxins [12]; however, no more than 20 potassium channel toxins have been characterized [13].

Despite the small number of neurotoxins that have been characterized up to date, potassium channel toxins are valuable tools for the investigation of the physiology, pharmacology, biochemistry and structure-function of K^+^ channels, the largest and most diverse family of ion channels. Among the K^+^ channel family, 15 subfamilies can be subdivided, according to their structure and function [14]. Of these different subfamilies, the voltage-gated potassium channel (K_V_) subfamily represents one of them and has an essential role in repolarizing the membrane after the initiation of an action potential [15]. They are also involved in physiological processes, such as regulation of heart rate, neuronal excitability, muscle contraction, neurotransmitter release, insulin secretion, Ca^2+^ signaling, cellular proliferation and migration and cell volume regulation [16,17].

Sea anemone K_V_ channel toxins can be divided into four structural classes according to structural differences and activity profile. Type 1 toxins inhibit *Shaker*-related K_V_ channel currents by a “functional dyad” directly interacting with the channel pore. These toxins were purified from the venom of sea anemones belonging to the *Actiniidae*, *Hormathiidae*, *Thalassianthidae* and *Stichodactylidae* families [13] and were exclusively characterized on mammalian K_V_ channels, using T-lymphocyte native currents, competitive binding experiments against ^125^I-dendrotoxins and different transfection cell expression systems [18,19]. Therefore, the biological meaning for the expression of these neurotoxins present in sea anemone venom still remains unknown. 

**Figure 1:**
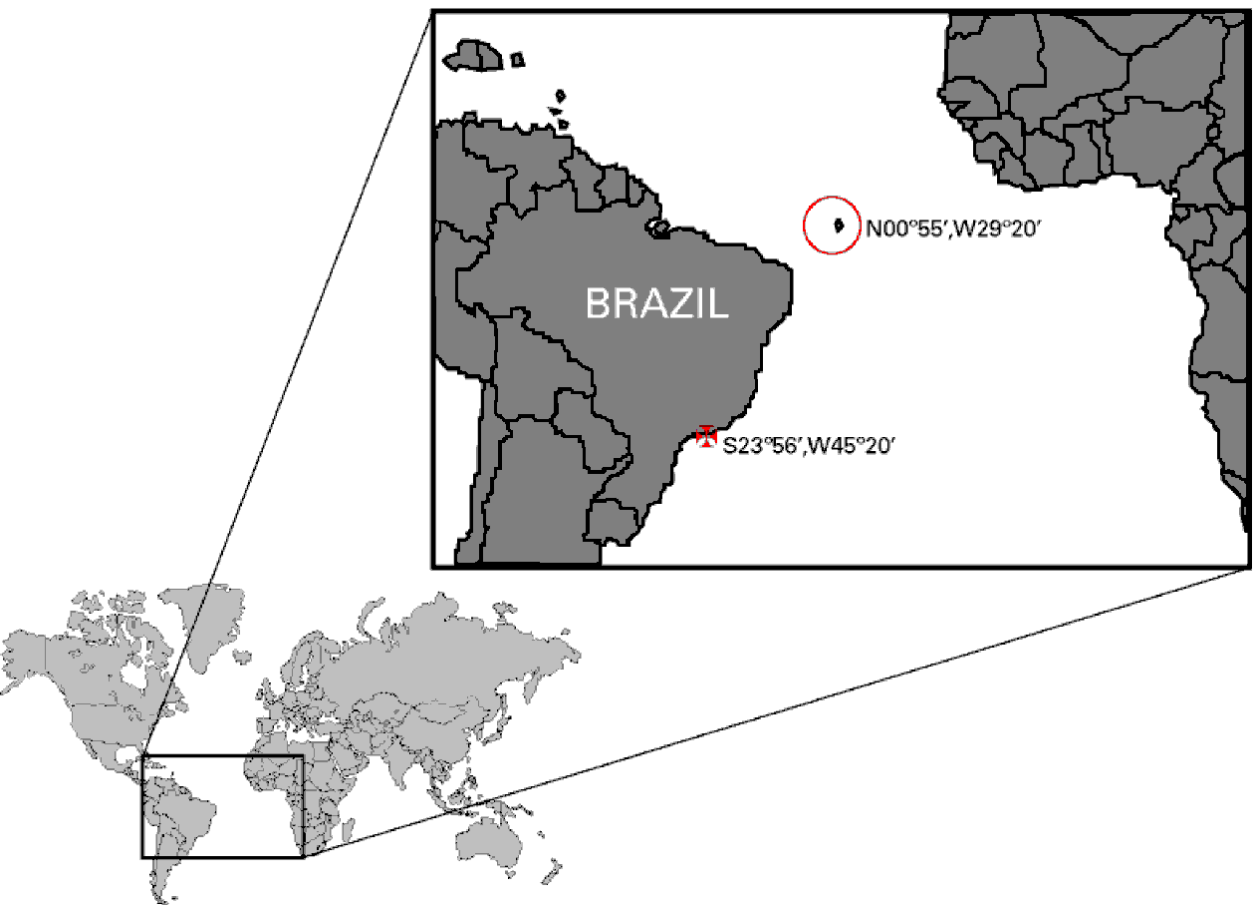
Map showing the geographic localization of the collection sites of *B. caissarum* populations used in this study. The red circle indicates Saint Peter and Saint Paul Archipelago (SPSPA) location at the North Atlantic Ocean (N0°55′; W29°20′). The Red Cross indicates the southeast coast of Brazil (São Sebastião beach—S23°56′, W45°20′), more than 4000 km distant from the SPSPA.

The sea anemone *Bunodosoma caissarum* (Correa, 1964) [20] is an endemic Brazilian species and can be found along the entire coastline and some oceanic islands [20,21]. Saint Peter and Saint Paul Archipelago (Saint Peter and Saint Paul Archipelago (SPSPA); N0°55′, W29°20′) is densely populated by this species, which is mostly found attached to the lower mid-littoral, as well as infra-littoral [22]. In this study, we report for the first time the characterization of the “neurotoxic fraction” from the venom of *B. caissarum* SPSPA population, and under the same experimental conditions, we compare it to the population found in the state of São Paulo littoral (southeast coast of Brazil; S23°56′, W45°20′) (Figure 1). Furthermore, we present the purification, biochemical analyses and electrophysiological characterization of two new type 1 sea anemone toxins, as well as their relationship with other known toxins based on sequence, structural and evolutionary analyses.

## 2. Results and Discussion

### 2.1. Venom Purification and Biochemical Characterization of BcsTx1 and BcsTx2

**Figure 2:**
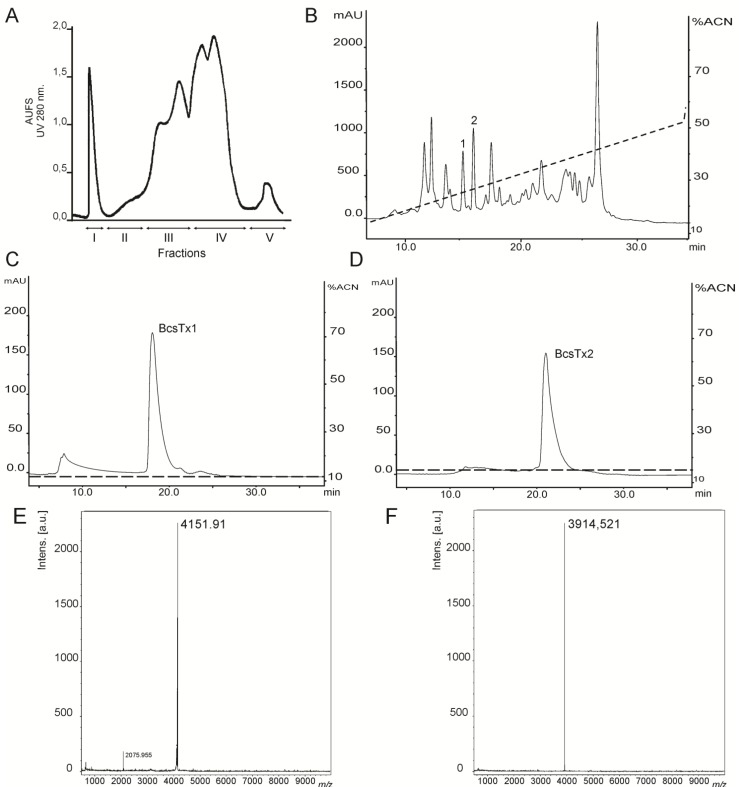
Isolation, purification and characterization of *B. caissarum* venom. (**A**) Gel-filtration chromatography of *B. caissarum* venom. Approximately 3.0 g of venom was injected into a Sephadex G-50 column, and the fractions were eluted with 0.1 M ammonium acetate buffer (pH 7.0). Fractions I to V were collected during UV (280 nm) monitoring. (**B**) Reverse-phase high performance liquid chromatography (rp-HPLC) chromatogram of Fraction III resulting from gel-filtration. The peptides from Fraction (Fr) III were eluted as described under the Experimental Section. Peaks labeled (1 and 2) were subjected to a second C18 rp-HPLC chromatography. (**C**) Peak 1 (BcsTx1) was purified on an analytical C18 column using an isocratic condition of 13% of acetonitrile containing 0.1% trifluoroacetic acid (TFA). (**D**) Purification of peak 2 (BcsTx2) using an isocratic condition of 16% of acetonitrile containing 0.1% TFA. (**E**) Mass measurement of purified BcsTx1 determined by Matrix-Assisted Laser Desorption/Ionization-Time Of Flight (MALDI-TOF), indicating a *m/z* of 2075.955 (*z* = 2) and 4151.91 (*z* = 1). (**F**) Mass spectrometry profile of purified BcsTx2 (*m/z* 3914.521).

Sea anemone venom extraction by electric stimulus provides a massive release of proteins, peptides and low molecular weight compounds from the nematocysts [23,24,25]. When this toxic mixture is applied to a Sephadex G-50 gel-filtration column, the peptide content of the venom is separated from enzymes, such as phospholipases and cytolysin [26,27,28]. Gel filtration of *B. caissarum* venom on Sephadex G-50 yielded five fractions named Fraction I to V (FrI–FrV) (Figure 2A), as previously described for the venom of *B. cangicum* [26] and *B. caissarum* population from the southeastern coast of Brazil [29]. Gel-filtration Fraction III (FrIII) from *B. caissarum* SPSPA population had the highest neurotoxicity when tested on swimming crabs (*Callinectes danae*) (data not shown), and it was further purified by reverse-phase high performance liquid chromatography (rp-HPLC) (Figure 2B). Elution peaks, labeled as 1 and 2 (Figure 2B), were able to fully block the insect channel *Shaker* IR and, thus, were subjected to a second purification step, leading to the pure toxins BcsTx1 and BcsTx2 (Figure 2C,D). Matrix-Assisted Laser Desorption/Ionization-Time Of Flight (MALDI-TOF) measurements of BcsTx1 and 2 generated an *m/z* data of 4151.91 and 3914.521, respectively (Figure 2E,F). These experimental masses correspond well with the theoretical molecular masses of 4151.93 Da of BcsTx1 and 3914.80 Da for BcsTx2.

Interestingly, the venom of *B. caissarum* population from the Southeastern coast of Brazil shows hemolytic activity and one actinoporin, named Bcs I, had been purified and biochemically characterized [30,31]. However, neither the whole venom, nor the fraction II (FrII) of SPSPA population (Figure 2A), showed cytolytic activity when tested on erythrocytes (data not shown). Up to date, cytolysin has been found in all classes of cnidarians, and more than 32 species of sea anemones have been reported to produce lethal cytolytic peptides [28,32]. Also, it has been shown that one species of sea anemone (e.g., *Actinia equina*) can produce more than one isoform, while others are devoid of any cytolytic activity (e.g., *Anemonia viridis*) [28,33]. Also, the incidence of cytolytic activity in corals (Anthozoa and Hydrozoa) is high, resembling the sea anemones, where cytolysins are widespread [32]. Gunthorpe and colleagues compared the bioactivity of aqueous extracts of scleractinian corals (Cnidaria, Anthozoa, Hexacorallia) from different families and concluded that the occurrence of cytolytic activity do not differ significantly among the genera and the species considered, except for the extracts of colonies of *Goniastrea australensis*, where intraspecific differences were found [34]. 

**Figure 3:**
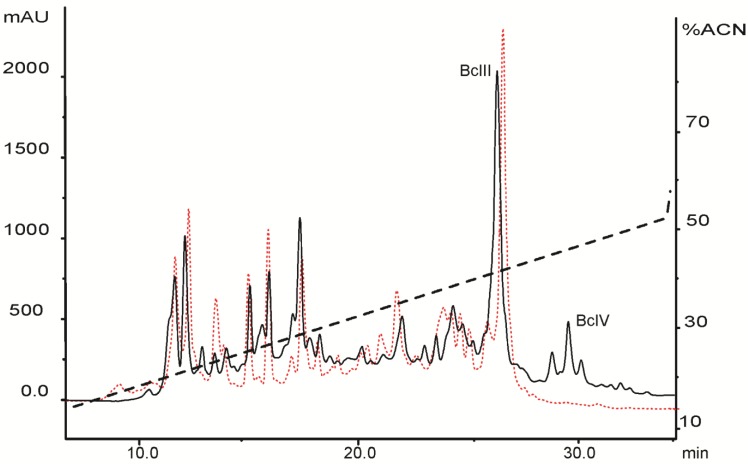
Comparison of the “neurotoxic fraction” (FrIII) from two populations of the sea anemone *B. caissarum*: southeastern coast of Brazil and Saint Peter and Saint Paul Archipelago. The black continuous line represents the rp-HPLC profile of FrIII from the Southeastern coast population. Labeled peaks were the previously characterized neurotoxins BcIII [29] and BcIV [35]. Red dotted line is the FrIII chromatographic profile of the SPSPA population. “*Neurotoxic fractions*” were submitted to rp-HPLC chromatography using a semi-preparative CAPCELL PAK C-18 column (1 × 25 cm, Shiseido Corp.), and their components were eluted with a linear gradient from 10% to 60% of acetonitrile containing 0.1% TFA, as described in the Experimental Section.

The rp-HPLC profile of fraction III (FrIII) of the SPSPA *B. caissarum* population yielded a very similar profile to that from the Southeastern coast of Brazil [35], suggesting that both populations releases a similar pattern of neurotoxic peptides (Figure 3). Until now, only two toxins from *B. caissarum* venom have been investigated: (i) BcIII that belongs to type 1 neurotoxins and bind at site 3 of the voltage-gated sodium channel (Na_V_), delaying the inactivation process [29], and (ii) BcIV, which does not have its exact target determined, yet. However, experiments using crab leg sensory nerve suggest a Na_V_-activity [35]. A superimposition of both rp–HPLC profiles of “neurotoxic fractions” from *B. caissarum* populations allows us to point out the following: (1) SPSPA sea anemone population has a BcIII-like toxin and (2), at the same retention time of BcIV, no elution peak is observed on the chromatographic profile of the SPSPA population. To our knowledge, such a degree of intraspecific variation in the peptide composition of sea anemone venom is novel.

Moran and colleagues [36,37] analyzed the evolution of a voltage-gated Na^+^ channel neurotoxin genes family from three genetically and geographically distinct populations of the sea anemone *Nematostella vectensis* [38,39] and from single specimens of *Actinia equina* and *Anemonia viridis*. Genomic data indicated much higher similarity among toxin genes within each species than to toxin genes of other species, suggesting that related neurotoxin genes family in sea anemones are subjected to a concerted evolution [40]. The authors also demonstrated that evolution driven by positive Darwinian selection would have occurred, as observed by the numerous substitutions in the putative neurotoxin genes from *A. equina* and *A. viridis*. 

Intraspecific diversity in the venom composition of various animal species, such as cone snails [41,42], bees [43], ants [44,45], spiders [46,47], scorpions [48,49] and snakes [50,51], have been reported using biochemical, pharmacological, proteomic and/or transcriptomic approaches. Abdel-Rahman and colleagues [52] used a combination of proteomic and biochemical assays to examine variations in the venom composition of the vermivorous *Conus vexillum* taken from two distinct geographical locations and concluded that the venom is highly diversified. Moreover, intraspecific variation in the peptides present in the venom from two species of fish-hunting cone snails (*C. striatus* and *C. catus*) has been reported. However, the venom compositions of individual snails of both species remained quite constant over time in captivity [42]. In contrast, proteomic analyzes of the venom of several specimens of a piscivorous cone snail (*C. consors*) revealed dramatic variations over time, which could be related to dynamics of peptide production by the secretory epithelium in the venom gland [53]. 

Similarly to cone snails, venom variability in specimens of *Tityus serrulatus* scorpion, collected within the same geographical area, has been shown. Specimens showed venom constituent variations, which were related to extraction events and to dynamics in gland production and peptide maturation [54,55]. Furthermore, investigation of intraspecific venom variation of four different populations of *Scorpio maurus palmatus* from geographically distant locations revealed highly significant differences among all populations and within each population studied. This may be due to geographic differential distribution of prey species, as well as their relative abundance in the environment [49,56]. Also, it has been demonstrated that ontogenetic variation of viperid snakes (Chordata, Reptilia, Viperidae) venoms could be related to differences between the feeding habits of juvenile and adult snakes, suggesting that variation in venom composition may reflect natural selection for greater efficiency in killing and digesting different prey types within the same location or in different locations [57,58,59]. 

Thus, the relationship between geographic distance and patterns of venom composition implicates spatial scale and localized ecological and genetic factors, such as gender, elapsed time after capture, dynamic expression of the gland and peptide maturation, genetic variation, environmental conditions, seasonality and geographical locations. In the current work, these factors were not standardized (except for venom collection and sea anemone size, presuming a similar age of specimens of each population), and additional studies will be necessary in order to assess more precisely these variations in venom composition and to enhance our understanding of the forces driving sea anemone venom evolution.

### 2.2. Amino Acid Sequences and Phylogenetic Analysis

**Figure 4:**
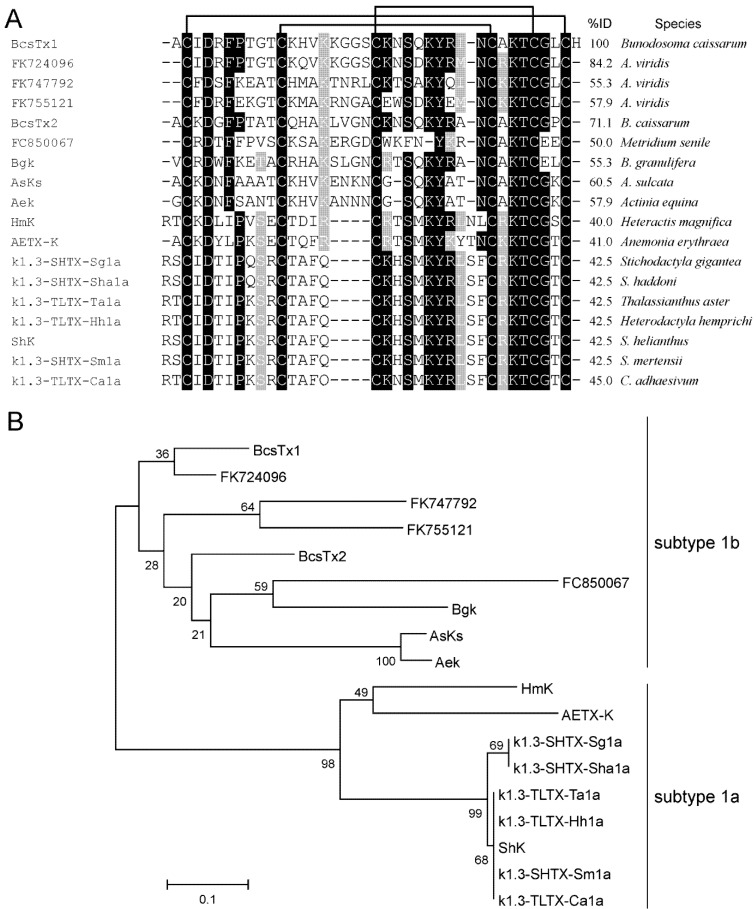
Phylogenetic analysis and sequence alignment. (**A**) Amino acid sequence of BcsTx1 and BcsTx2 and multiple sequence alignment with the other members of type 1 sea anemone toxins. Alignment was based on the cysteine residues. Disulfide bridge pattern are indicated. Amino acid identities (black boxes) and similarities (grey boxes) are shown. (**B**) The phylogenetic tree of type 1 sea anemone K_V_-toxins was constructed with the *Neighbor*-*joining* algorithm of MEGA 4.0. The consensus tree shown supports the suggested division of sea anemone type 1 into two different subtypes. The scale bar shows amino acid substitution rates. Only the mature region of the sequences reported as FC850067, FK724096, FK755121 and FK747792 were used in the analysis.

The native and non-reduced toxins were directly sequenced by automated Edman degradation, which gave unequivocal amino acid sequences. Cysteines were assumed as blank cycles. Sequences similarity indicated that BcsTx-1 and -2 are new members of the type 1 sea anemone toxins, acting on voltage-gated potassium channels (K_V_), which also include the peptides BgK (*Bunodosoma granulifera*) [60], ShK (*Stichodactyla helianthus*) [61], HmK (*Heteractis magnifica*) [62], AsKS (*Anemonia viridis*) [63], AeK (*Actinia equina*) [64], AETxK (*A. erythraea*) [65], κ1.3-SHTX-Sha1a (*S. haddoni*), κ1.3-TLTX-Ca1a (*Cryptodendrum adhaesivum*), κ1.3-TLTX-Hh1a (*Heterodactyla hemprichi*), κ1.3-SHTX-Sg1a (*S. gigantea*), κ1.3-SHTX-Sm1a (*S. mertensii*), κ1.3-TLTX-Ta1a (*Thalassianthus aster*) [13], FC850067 (*Metridium senile*), FK724096, FK755121 and FK747792 (*Anemonia viridis*) [66] (Figure 4A). The sequences reported as FC850067, FK724096, FK755121 and FK747792 are the Expressed Sequence Tags (ESTs) accession numbers of deduced mature peptide sequences from translated nucleotides of the above mentioned species.

Members of the type 1 have 35–38 amino acid residues and three disulfide bridges are paired as C1–C6, C2–C4 and C3–C5, by similarity. Toxins are moderately conserved, all sharing 39.5%−100% sequence similarity and, thus, can be further divided into subtype 1a, which has four amino acids between the second and third Cys residues from the *N*-terminus, and subtype 1b, with eight amino acids [13,65]. BcsTx-1 and -2, together with toxins BgK (from *B. granulifera*), AsKs (*Anemonia viridis*), AeK (*Actina equina*) and the four sequences of the mature portions of the putative toxins (from *A. viridis* and *Metridium senile*), are members of subtype 1b toxins (Figure 4B). Subtype 1a is composed by nine toxins (HmK, AETX-K, ShK, κ1.3-SHTX-Sha1a, κ1.3-TLTX-Ca1a, κ1.3-TLTX-Hh1a, κ1.3-SHTX-Sg1a, κ1.3-SHTX-Sm1a and κ1.3-TLTX-Ta1a) that share more than 80% sequence identity with one another (Figure 4B). Type 1 toxins block potassium currents of *Shaker* and *Shaw* subfamilies of K_V_ channels and also block the intermediate conductance calcium-activated K^+^ channels, and they can differ markedly in potency or selectivity [1]. Moreover, all peptides possess a conserved functional core composed of a key basic residue (lysine) associated with a 6.6 ± 1 Å distant key aromatic residue (tyrosine) [67]. The side chain of the lysine residue enters the ion channel pore and is surrounded by four asparagine residues of the selective filter of the channel. The key aromatic residue will interact through both electrostatic forces and hydrogen bonding with a cluster of aromatic residues in the P-loop region [68,69]. 

**Figure 5:**
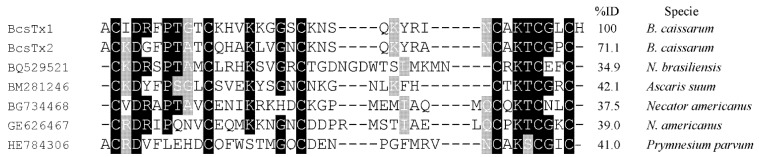
Alignment. Amino acid sequences of BcsTx1 and BcsTx2 were aligned with part of the mature portion of the putative proteins from *Ascaris suum* (Nematoda, Secernentea) (GenBank # BM281246), *Necator americanus* (Nematoda, Rhabditea) (GenBank # BG734468 and GE626467) and *Nippostrongylus brasiliensis* (Nematoda, Secernentea) (GenBank # BQ529521) after conducting a BLAST homology search of the Expressed Sequence Tags (ESTs) on databases.

Interestingly, type 1 sea anemone toxins could be classified as belonging to the six-cysteine (SXC) protein domain, whose first members were identified in surface coat components of the dog ascaridid *Toxocara canis* (Nematoda, Secernentea) and, later, also have been identified in many additional nematodes [70,71]. This domain is composed of short (36 to 42 amino acids) peptides, with six conserved cysteines, that can be found in many parasitic nematodes, such as *Ascaris suum* and *Necator americanus* [72]. The physiological role of these peptides has not been established, yet; however, it is believed that they might interfere with the local and systemic immune system and with gut muscles of the host [73]. As already mentioned, sea anemone type 1 toxins possess a conserved “functional dyad” motif, which is not universally present in nematodes (Figure 5). However, if we observe the basic and aromatic residues (lysine and phenylalanine) of the putative protein from *Ascaris suum*, we might suggest a possible K_V_ channel blocker activity. Thus, considering that, throughout evolution, proteins found in venoms are the result of toxin recruitment events in which a protein gene involved in a regulatory process is duplicated and the new gene is selectively expressed in the venom apparatus [74], we may suppose that the existence of the SXC domain in different phyla reflect their common ancestry.

### 2.3. BcsTx1 and BcsTx2 Pharmacological Profiles

**Figure 6:**
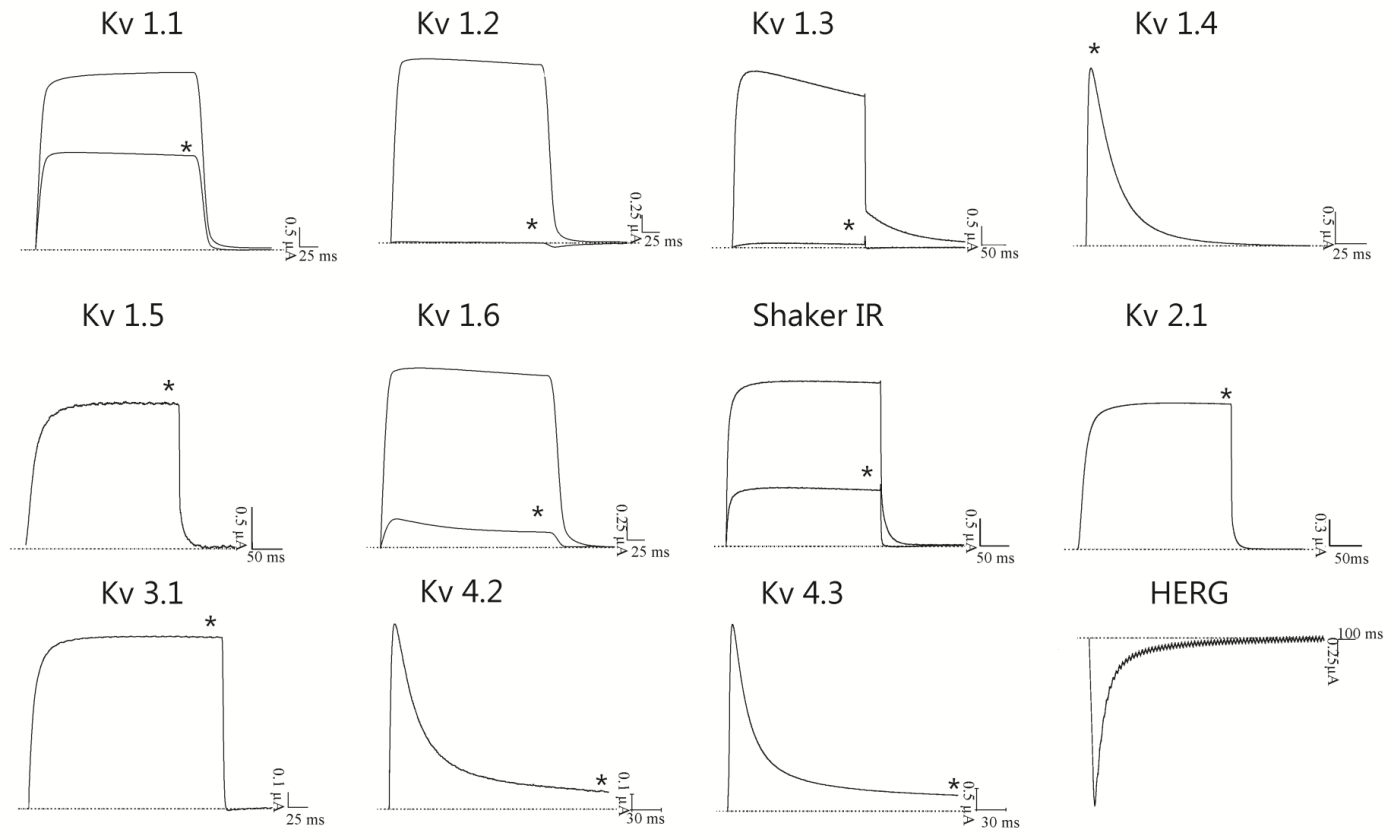
Electrophysiological screening of BcsTx1 (0.5 µM) on several cloned voltage–gated potassium channel isoforms belonging to different subfamilies. Representative traces under control and after application of 0.5 µM of BcsTx1 are shown. The * indicates steady-state current traces after toxin application. The dotted line indicates the zero-current level. This screening shows that BcsTx1 selectively blocks K_V_1.x channels at a concentration of 0.5 µM.

**Figure 7:**
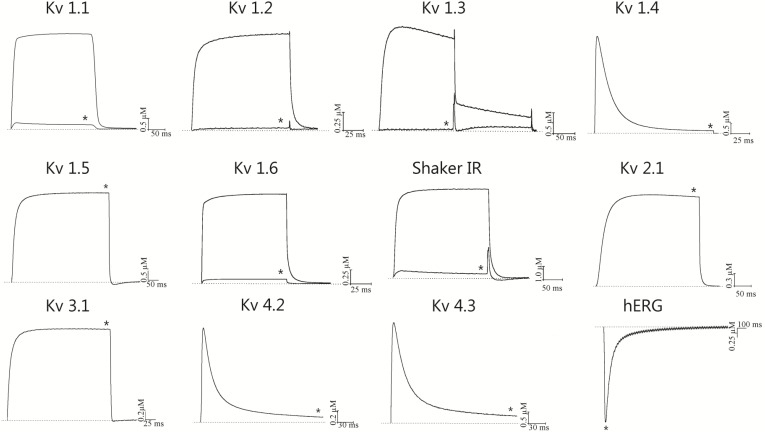
Inhibitory effects of BcsTx2 (3 µM) on 12 voltage-gated potassium channels isoforms expressed in *X. laevis* oocytes. Representative whole-cell current traces in the absence and in the presence of 3 µM BcsTx2 are shown for each channel. The dotted line indicates the zero-current level. The * indicates steady state current traces after application of 3 µM BcsTx2. This screening carried out on a large number of K_V_ channel isoforms belonging to different subfamilies shows that BcsTx2 selectively blocks *Shaker* channels subfamily.

Sequence alignment and phylogenetic analysis (Figure 4A,B) indicated that BcsTx-1 and -2 are new members of the type 1 (subtype 1b) toxins from sea anemones that are known to be potent inhibitors of K_V_ channels. The pharmacological profile of BcsTx-1 and -2 were determined on a wide range of twelve K_V_ channels (rK_V_1.1, rK_V_1.2, hK_V_1.3, rK_V_1.4, rK_V_1.5, rK_V_1.6, rK_V_2.1, rK_V_3.1, rK_V_4.2, rK_V_4.3, *h*ERG and the insect channel *Shaker* IR; r: rat and h: human). Channels were expressed in *X. laevis* oocytes, and their currents were recorded by using two-electrode voltage-clamp technique. BcsTx1 (0.5 µM) inhibited rKv1.1, rKv1.2, rKv1.3, rKv1.6 and *Shaker* IR channels with 44% ± 2%, 100%, 100%, 88% ± 3% and 64% ± 4%, respectively (Figure 6). BcsTx2 (3 µM) showed an effect on potassium currents inhibiting 96% ± 2.1%, 100%, 100%, 98% ± 1.75% and 94% ± 2% of rK_V_1.1, rK_V_1.2, hK_V_1.3, rK_V_1.6 and *Shaker* IR, respectively (Figure 7). Type 1 toxins, such as BgK and ShK, have been extensively characterized. BgK was found to block K_V_1.1-3 and K_V_1.6 channels with potencies in the nanomolar range [60]. ShK was originally found to block K_V_1.3 channels [69,75], but also blocks K_V_1.1-4 and K_V_1.6 [61,76]; and more recently, it has been found that ShK shows activity against K_V_3.2 channels [77]. Both BgK and ShK block intermediate conductance K (Ca) channels [78]. Some of the other type 1 toxins were indirectly assayed by competitive inhibition of the binding of ^125^I-dendrotoxins, allowing the conclusion that they will show activity on K_V_1.1, K_V_1.2 and/or K_V_1.6, since dendrotoxins only block the current of these K_V_ channels. The AsKs toxin has been characterized as a blocker of K_V_1.2 current expressed in *Xenopus* oocytes, and no biological activity has been reported to FC850067, FK724096, FK755121 and FK747792 [13,62,63,64,65]. Thus, it is worth mentioning that our work represents the first electrophysiological characterization of type 1 sea anemone toxin activity on cloned *Shaker* IR insect channel. 

**Figure 8:**
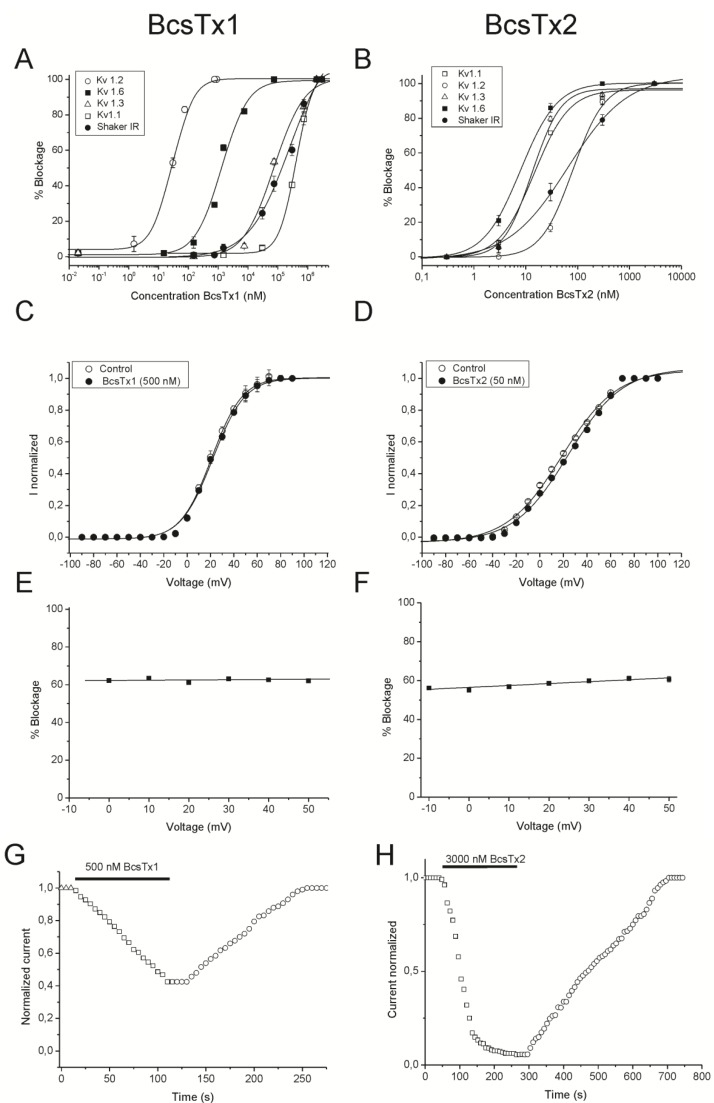
Functional features of BcsTx1 and BcsTx2 on K_V_ channels. (**A**, **B**) Dose-response curves of BcsTx1 and BcsTx2 on rK_V_1.1, rK_V_1.2, hK_V_1.3, rK_V_1.6 and *Shaker* IR channels. The curves were obtained by plotting the percentage blocked current as a function of increasing toxin concentrations. All data are presented as the mean ± standard error (*n* ≥ 3). (**C**, **D**) Current-voltage relationship for *Shaker* IR isoform in control condition and in the presence of BcsTx1 (500 nM) and BcsTx2 (50 nM). Current traces were evoked by 10 mV depolarization steps from a holding potential of −90 mV. Open circles indicates the V_1/2_ in control; closed circles indicate the addition of toxins. (**E**, **F**) Percentage of currents left after application of BcsTx1 (500 nM) and BcsTx2 (50 nM) on *Shaker* IR channel. In a range of test potentials from −10 mV to +50 mV, no difference was observed in the degree of BcsTx1- and BcsTx2-induced blockage. (**G**, **H**) Representative experiment of the time course of Shaker IR current inhibition with BcsTx1 (500 nM) and BcsTx2 (3000 nM) and the reversibility hereof. Control (open square); washout (open circles). Blockage occurred rapidly, and binding was reversible upon washout. Plots shown are a representative of at least three individual experiments.

In order to characterize the potency and selectivity profile, concentration-response curves were constructed for BcsTx1. IC_50_ values yielded 405 ± 20.56 nanomolar (nM) for rKv1.1, 0.03 ± 0.006 nM for rKv1.2, 74.11 ± 20.24 nM for hKv1.3, 1.31 ± 0.20 nM for rKv1.6 and 247.69 ± 95.97 nM for *Shaker* IR (Figure 8A and Table 1). A concentration-response curve was also constructed to determine the concentration at which BcsTx2 blocked half of the channels. The IC_50_ values calculated are 14.42 ± 2.61 nM for rK_V_1.1, 80.40 ± 1.44 nM for rK_V_1.2, 13.12 ± 3.29 nM for hK_V_1.3, 7.76 ± 1.90 nM for rK_V_1.6 and 49.14 ± 3.44 nM for *Shaker* IR (Figure 8B and Table 1). Similar to BgK, the BcsTx-1 and -2 potencies are within the nanomolar range and are more potent when compared to type 2 sea anemone toxins, such as kalicludines (AsKC1-3), which blocks K_V_1.2 channels with IC_50_ values around 1 µM [63]. In general, previous work has shown that type 1 sea anemone toxins are more potent than type 2, and it has been proposed in the literature that toxins with a “functional dyad” are more potent, because it provides a secondary anchoring point, contributing to a higher toxin affinity [68,79]. However, APEKTx1, a type 2 toxin from *A. elegantissima*, is a selective blocker of K_V_1.1, with an IC_50_ value of 1 nM, and the existence of a “functional dyad” has not been shown [80]. Moreover, the electrophysiological characterization of the scorpion toxins Pi1 and Tc32 (from *Pandinus imperator* and *Tityus cambridgei*, respectively), which are known to potently inhibit K_V_1 channels, suggested that other amino acids, rather than those of the “functional dyad”, are also involved in both potency and selectivity of the K_V_ channel isoforms [81,82]. Although, it is worth noting that the “functional dyad” of α-KTx family of scorpion toxins is very important for high affinity block and selectivity [83]. For instance, toxin Pi2 (α-KTx7.1), from the venom of *P. imperator*, has a “functional dyad” formed by Lys27 and Trp8 and is able to block K_V_1.2 current with an IC_50_ value (0.032 nM) comparable to BcsTx1 [84]. Also, MgTX (α-KTx2.2) toxin, from *Centruroides margaritatus*, binds with very high affinity to K_V_1.6 (IC_50_ value of 5 nM), and the role of the side chain of the dyad lysine (Lys27) as a critical residue to the binding of the toxin to the ion conduction pathway of the channel was proposed [85].

In order to elucidate whether BcsTx-1 and -2 block the current through a physical obstruction of the *Shaker* IR channel pore or act as gating modifiers, current-voltage (I-V) experiments were performed. The currents were inhibited at the test potentials from −90 to 100 mV, and the inhibition was not associated with a change of the shape of the I-V relationship. The control curve and the curve in the presence of BcsTx1 (500 nM) were characterized by a V_1/2_ values of 20.85 ± 0.69 mV and 22.62 ± 0.73 mV, respectively. Moreover, the control curve and the curve in the presence of BcsTx2 (50 nM) were characterized by a V_1/2_ values of 18.49 ± 1.49 mV and 23.88 ± 1.57 mV, respectively. The V_1/2_ of activation was not significantly shifted (*p* < 0.05), and thus, channel gating was not altered by BcsTx1 and BcsTx2 binding (Figure 8C,D). Additionally, BcsTx-1 and -2 shows a non-dependence of voltage for the blockage on a wide range from −10 mV to 50 mV (Figure 8E,F); the blockage effect was reversible, and a complete recovery was observed after washout, suggesting an extracellular site of action (Figure 8G,H). To date, type 1 sea anemone toxins have been described to act solely through a K_V_ channel pore-blocking mechanism [1].

**Table 1:** BcsTx1 and BcsTx2 IC_50_ values in nanomolar (nM).

Isoforms	BcsTx1	BcsTx2
**K_V_1.1**	405 ± 20.56	14.42 ± 2.61
**K_V_1.2**	0.03 ± 0.006	80.40 ± 1.44
**K_V_1.3**	74.11 ± 20.24	13.12 ± 3.29
**K_V_1.6**	1.31 ± 0.20	7.76 ± 1.90
***Shaker* IR**	247.69 ± 95.97	49.14 ± 3.44

### 2.4. Bioinformatics Analysis

#### Molecular Models of BcsTx-1 and -2

**Figure 9:**
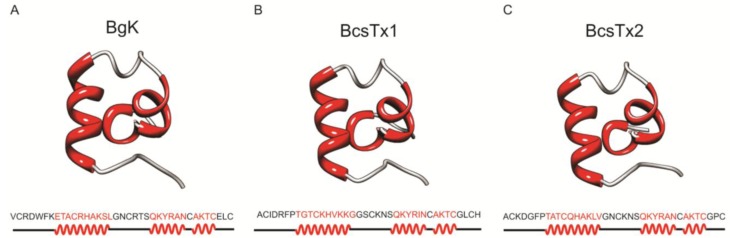
3-D model representation of BcsTx1 and BcsTx2. Models were constructed using BgK toxin as template (Protein Data Bank (PDB) code 1BGK). (**A**) Ribbon representation of nuclear magnetic resonance (NMR) structure of BgK. Amino acid sequence and secondary structure: α-helix (red) and loops (gray). (**B**) Stereoscopic 3-D model of BcsTx1. (**C**) BcsTx2 molecular model.

Venomous animals produce a wide variety of neurotoxins with different types of amino acid sequences, secondary structures and disulfide bridge frameworks, and none of them is definitively associated with a particular animal species or ion channel selectivity [79]. Type 1 sea anemone toxins are associated with the αα type of family fold. BgK toxin has a “helical cross-like” motif in which one α-helix is disposed perpendicular to the others [67] (Figure 9A) and ShK has a “helical capping” motif (3_10_αα), since one α-helix (formed by three amino acid residues) caps the other two helical structures [86]. The molecular models of BcsTx-1 and -2 (Figure 9B,C) were constructed using BgK as template, and the quality of the models were analyzed using PROCHECK [87]. BcsTx-1 and -2 share 55.3% and 62% of sequence identity with BgK, respectively. BcsTx1 and BcsTx2 analyses revealed that 87.1% and 90.0% of residues are located in the most favored regions, 12.9% and 6.7% are located in additionally allowed regions and 0% and 3.3% are located in generously allowed regions of the Ramachandran diagram, respectively [88]. The secondary structure of both toxins consists of three α-helical segments; the first α-helix comprises the amino acids 8–17, the second comprises the residues 24–29 and the third α-helix consists of the amino acids 31–34. Despite the overall moderate identity between these three toxins, the residues of the second and third α-helices are highly identical. BgK second α-helix shares 83.3% and 100% of identity to BcsTx-1 and -2, respectively, and the third is 100% identical within the three toxins.

## 3. Experimental Section

### 3.1. Sea Anemone Collection, Venom Isolation and Neurotoxins Purification

Specimens of the sea anemone *Bunodosoma caissarum* (3.5–4.0 cm of diameter) were collected at the Saint Peter and Saint Paul Archipelago (N0°55′, W29°20′), Brazil. The sea anemones were maintained in aquarium for 24 h, and then the venom was obtained by electrical stimulation of the animals, according to the method of Malpezzi *et al.* [23]. The venom was fractionated first by gel-filtration chromatography using a Sephadex G-50 column (1.9 × 131 cm, GE Healthcare, Uppsala, Sweden), and afterwards, the fraction containing the neurotoxic peptides was submitted to reverse-phase HPLC chromatography in an ÄKTA Purifier system (GE Healthcare, Uppsala, Sweden) using a semi-preparative CAPCELL PAK C-18 column (1 × 25 cm, Shiseido Corp., Kyoto, Japan). Elution was done in a linear gradient from 10% to 60% of acetonitrile containing 0.1% TFA at a flow rate of 2.5 mL/min during 40 min, and the peptides were monitored at UV 214 nm. Pure BcsTx1 and BcsTx2 were obtained using an analytical CAPCELL PAK C-18 column (0.46 × 15 cm, Shiseido Corp., Kyoto, Japan) and different gradients of the solvent described above, at a flow rate of 1 mL/min. The protein content of the pure peptides was estimated by the bicinchoninic acid assay (BCA) method (Pierce, Rockford, IL, USA).

### 3.2. Mass Spectrometry Analysis

Mass spectrometry analyses were performed on an Ultraflex II TOF/TOF MALDI (Bruker Daltonics, Bremen, Germany) equipped with Nd-YAG Smartbeam laser (MLN 202, LTB) under reflectron mode. The laser frequency was adjusted to 50 Hz. The matrix, α-cyano-4-hydroxycinnamic acid (Sigma-Aldrich Co., St. Louis, MO, USA), was prepared at a concentration of 20 mg/mL in 1:1 acetonitrile containing 0.1% TFA solution. External calibration was performed using peptide calibration standard II (Bruker Daltonics, Bremen, Germany). Sample solution (1 μL) dropped onto the MALDI sample plate was added to the matrix solution (1 μL) and dried at room temperature. Data were analyzed using the FlexAnalysis 3.0 program (Bruker Daltonics, Bremen, Germany).

### 3.3. Amino Acid Sequence Determination

Samples of the native peptides (BcsTx1 and BcsTx2) (50–200 pmol) were sequenced by Edman degradation using the automated PPSQ-33A protein sequencers (Shimadzu, Kyoto, Japan) coupled to reverse phase separation of phenylthiohydantoin (PTH)-amino acids on a WAKOSIL-PTH (4.6 × 250 mm) column (Wako, Osaka, Japan), according to the manufacturer’s instructions.

### 3.4. Expression of Voltage-Gated Ion Channels in *Xenopus laevis* Oocytes

Stage V–VI of *X. laevis* oocytes were harvested by partial ovariectomy under anesthesia (3-aminobenzoic acid ethyl ester methanesulfonate salt, 0.5 g/L from Sigma-Aldrich Co., Saint Louis, MO, USA). The oocytes were defolliculated for 2 h by treatment with 2 mg/mL collagenase (Sigma-Aldrich Co., Saint Louis, MO, USA) in Ca^2+^ free ND96 solution (in mM: 96 NaCl; 2 KCl; 1 MgCl_2_; 5 HEPES adjusted pH 7.4). For the expression of K_V_ channels (K_V_1.1–K_V_1.6, K_V_2.1, K_V_3.1, K_V_4.2, K_V_4.3, *h*ERG and the insect channel *Shaker* IR), the linearized plasmids were transcribed using the T7 or SP6 mMessage-mMachine transcription kit (Ambion, Austin, TX, USA). Oocytes were injected with 50 nL of cRNA at a concentration of 1 ng/nL using a microinjector (Drummond Scientific, Broomall, PA, USA). The oocytes were maintained in a ND96 solution (in mM: 96 NaCl, 2 KCl, 1.8 CaCl_2_, 2 MgCl_2_ and 5 HEPES; adjusted pH 7.4), supplemented with 50 µg/mL gentamicin sulfate.

### 3.5. Electrophysiological Recordings

Two-electrode voltage-clamp recordings were performed at room temperature (18–22 °C) using a Geneclamp 500 amplifier (Molecular Devices, Sunnyvale, CA, USA) controlled by a pClamp data acquisition system (Axon Instruments, Union City, CA, USA). Whole-cell currents from oocytes were recorded from 1 to 3 days after injection. Bath solution was the same ND96 solution described above. Voltage and current electrodes were filled with 3 M KCl. Resistances of both electrodes were kept between 0.8 and 1.0 ΩM. The elicited currents were filtered at 500 Hz using a four-pole lowpass Bessel filter. Leak subtraction was performed using a −P/4 protocol. K_V_1.1–K_V_1.6 and *Shaker* IR currents were evoked by 500 ms depolarizations to 0 mV, followed by a 500 ms pulse to −50 mV, from a holding potential of −90 mV. Current traces of *h*ERG channels were elicited by applying a +40 mV prepulse for 2 s, followed by a step to −120 mV for 2 s. K_V_3.1, K_V_ 4.2 and K_V_4.3 currents were elicited by 500 ms pulses to +20 mV from a holding potential of −90 mV. To assess the concentration-response relationships, data were fitted with the Hill equation:

*y* = 100/[1 + (EC50/[toxin])*h*] (1)

where *y* is the amplitude of the toxin-induced effect, EC_50_ is the toxin concentration at half maximal efficacy, [toxin] is the toxin concentration and *h* is the Hill coefficient. In order to investigate the current-voltage relationship, current traces were evoked by 10 mV depolarization steps from a holding potential of −90 mV. The activation curves were fitted with a Boltzmann relationship of the form:

1/(1 + *e*^[−(V − V^^1/2)/^*^S^*^]^) (2)

where V_1/2_ is the voltage for half-maximal activation and *s* is the slope factor. The activation kinetics were obtained by mono-exponential fits to the raw current traces. 

### 3.6. Phylogenetic Analysis and Sequence Alignment

The functional dendrogram reported here was constructed using the N*eighbor-joining* method [89] of the publicly available software MEGA4 [90]. A multiple sequence alignment of BcsTx-1 and -2 and sea anemone type 1 voltage-gated potassium channel toxins was done with ClustalW2 (http://www.ebi.ac.uk/Tools/msa/clustalw2 [91]). Sequences analyzed were that of Aek (=Swiss-Prot # P81897) from the venom of the sea anemone *Actinia equina* [64], AETX-K (=Swiss-Prot # Q0EAE5) from *Anemonia erythraea* [65], AsKs (=Swiss-Prot # Q9TWG1), from *Anemonia sulcata* [63], Bgk (=Swiss-Prot # P29186) from *Bunodosoma granulifera* [60], HmK (=Swiss-Prot # O16846) from *Radianthus magnifica* [62], κ1.3-SHTX-Sha1a (=GenBank # AB595205) from *Stichodactyla haddoni* [13,92], κ1.3-TLTX-Ca1a (=GenBank # AB595207) (*Cryptodendrum adhaesivum*), κ1.3-TLTX-Hh1a (=GenBank # AB595208) (*Heterodactyla hemprichi*), κ1.3-SHTX-Sg1a (=GenBank # AB595204) (*Stichodactyla gigantea*), κ1.3-SHTX-Sm1a (=GenBank # AB595206) (*Stichodactyla mertensii*) and κ1.3-TLTX-Ta1a (=GenBank # AB595209) (*Thalassianthus aster*) [13], ShK (=Swiss-Prot # P29187) from *Stichodactyla helianthus* [61], FK724096, FK755121 and FK747792 from *Anemonia viridis* [66] and FC850067 from *Metridium senile*. The tree shown is a bootstrap consensus based upon 1000 replications of the *Neighbor*-*joining* algorithm with Poisson correction. Numbers are bootstrap percentages.

### 3.7. Structure Computational Modeling

3D-structures of *B. caissarum* toxins were modeled using the publicly available program MODELLER9v10 [93]. BcsTx-1 and -2 were modeled using as template BgK, a voltage-gated potassium channel toxin from the venom of the sea anemones *Bunodosoma granulifera* (PDB code: 1BGK). Models were refined based on predicted secondary structure using SCRATCH Protein Predictor [94] and PROCHECK [87].

### 3.8. Statistical Assessment

Comparison of two sample means was made using a paired Student’s *t* test (*p <* 0.05). All data represent at least three independent experiments (*n* ≥ 3) and are presented as the mean ± standard error. All data were analyzed using Clampfit 10.3 (Molecular Devices, Sunnyvale, CA, USA) and Origin 7.5 software (Origin Lab., Northampton, MA, USA). 

## 4. Conclusions

In summary, we demonstrate, for the first time, a venom composition and biological activity comparison between two geographically distant populations of sea anemones. Moreover, this is the first electrophysiological characterization of a sea anemone type 1 toxin on cloned *Shaker* IR insect channels, allowing us to suggest that the role of these toxins in the physiology of the sea anemone would be related with predation and defense against predators and highlights the possible application of these peptides as tools for research in neuroscience, as well as in the development of novel insecticides.
